# Increased MLKL mRNA level in the PBMCs is correlated with autoantibody production, renal involvement, and SLE disease activity

**DOI:** 10.1186/s13075-020-02332-7

**Published:** 2020-10-14

**Authors:** Mingjiao Zhang, Hongyu Jie, Yong Wu, Xinai Han, Xing Li, Yi He, Xingliang Shi, Yuwei Luo, Ying Sun, Jinlong Yang, Jing Yang, Shulv Quan, Xiaobin Lao, Liping Tan, Erwei Sun

**Affiliations:** 1 Department of Rheumatology and Immunology, The Third Affiliated Hospital, Southern Medical University, Guangzhou, China; 2 Guangdong Provincial Key Laboratory of Bone and Joint Degeneration Diseases, The Third Affiliated Hospital, Southern Medical University, Guangzhou, China; 3 Clinical Medical Laboratory Center, The First Affiliated Hospital, Jinan University, Guangzhou, China; 4 Department of Rheumatology and Immunology, Shunde Hospital, Southern Medical University, Guangzhou, China

**Keywords:** SLE, Necroptosis, MLKL, mRNA, PBMCs, Diagnosis

## Abstract

**Background:**

Necroptosis is a form of regulated necrosis that is involved in various autoimmune diseases. Mixed lineage kinase domain-like pseudokinase (MLKL) has been identified as a key executor of necroptosis; however, the significance of MLKL in peripheral blood mononuclear cells (PBMCs) of systemic lupus erythematosus (SLE) has not been investigated. In this study, we aimed to determine the mRNA level of MLKL in PBMCs and examine its relationship with clinical features and serological parameters in SLE.

**Methods:**

Real-time transcription-polymerase chain reaction (RT-PCR) analysis was used to determine the expression of MLKL mRNA in PBMCs from 59 patients with SLE, 25 patients with rheumatoid arthritis (RA), and 30 age- and sex-matched healthy controls (HC). Spearman’s correlation test was performed to assess the correlation of MLKL mRNA with clinical variables. The receiver operating characteristic (ROC) curve was created to evaluate the diagnostic value.

**Results:**

Our results showed MLKL mRNA in PBMCs was upregulated in SLE patients compared to that in RA and HC individuals. SLE patients positive for antinuclear antibodies had significantly higher MLKL mRNA than antibody-negative patients. In SLE patients, MLKL mRNA was found to be upregulated in patients with lupus nephritis (LN) as compared with patients without LN, and also higher in active patients than in stable patients. MLKL mRNA level was significantly and positively correlated with c-reaction protein (CRP) (*r* = 0.3577, *p* = 0.0237), erythrocyte sedimentation rate (ESR) (*r* = 0.4091, *p* = 0.0043), serum immunoglobulin G (IgG) concentration (*r* = 0.3546, *p* = 0.0289), and the numbers of positive antinuclear antibodies (ANAs) (*r* = 0.3945, *p* = 0.0432). ROC analysis showed that MLKL mRNA in PBMCs had an area under the curve of 0.9277 (95% CI 0.8779–0.9775, *p* < 0.001) to discriminate SLE from controls.

**Conclusions:**

These results suggest that increased MLKL mRNA level in the PBMCs of SLE patients is correlated with renal involvement and disease activity, identifying a subgroup of patients with SLE or LN who may benefit from early diagnosis and therapies targeting MLKL.

## Background

SLE is a complex, heterogeneous systemic autoimmune disease that attacks various cells and tissues, resulting in chronic inflammation and persistent tissue damage [1]. A notable characteristic of SLE is the production of pathogenic autoantibodies recognizing nucleic acids or proteins binding to nucleic acids [2]. Dysregulated cell death processes and defective clearance of dying cells have been proposed to contribute to autoantigen generation and induction of autoantibodies, as well as other aberrant immune responses in SLE [3].

Necroptosis is a special form of necrosis that is triggered by multiple pathways [4]. In cells where caspase-8 is inhibited, inflammatory signaling via tumor necrosis factor (TNF) super family receptors, interferons (IFNs), toll-like receptor 3 (TLR3), or TLR4 can lead to the phosphorylation of receptor-interacting serine/threonine-protein kinase 1 (RIPK1), RIPK3, and MLKL [5–8]. The phosphorylated MLKL inserts itself into the cell membrane, disrupts its integrity, and leads to cell death [9]. Various studies have revealed that necroptosis could be implicated in the pathogenesis of many inflammatory and autoimmune diseases, including SLE [6, 10, 11].

The diversity of the SLE might reflect differences in pathogenesis between different subgroups [12]. Approaches are needed to better understand the pathogenesis and to find new targets for various stages of the disease. Considering the role of necroptosis in the pathogenesis and development of SLE [13–16], we aimed to analyze MLKL mRNA of PBMCs and figure out whether it could serve as a biomarker for disease diagnosis and monitoring.

## Subjects and methods

### Study cohorts

We enrolled 59 patients with SLE and 25 patients with RA admitted to the Department of Rheumatology and Immunology of the Third Affiliated Hospital, Southern Medical University, China, from July 2019 to December 2019. Thirty age- and sex-matched HC individuals with no history of SLE or other immune disorders were enrolled at the Health Management Center in the same hospital. All the subjects had no infections. The diagnosis of SLE was according to the 1997 revised American College of Rheumatology (ACR) classification criteria [17]. All participants provided written informed consent for blood draw and MLKL mRNA testing. Serum samples were obtained from all participants during the study.

Analyzing subgroups of SLE is increasingly important to better understand the pathogenesis of disease and provide more tailored medic protocols. Then, we sorted SLE patients into different groups based on serological features, renal involvement, and disease activity. Firstly, SLE patients were divided into two groups: positive ANA group (*n* = 48) and negative ANA group (*n* = 11). Another variable was renal involvement, defined as fulfilling the ACR classification criteria for renal manifestation of SLE (≥ 0.5 g of proteinuria per day or 3+ protein on urine dipstick analysis) or having evidence of LN on kidney biopsy. SLE patients were divided into two groups: LN patients (*n* = 23) and non-LN (*n* = 36) patients. Lastly, SLE patients were evaluated using the SLE Disease Activity Index (SLEDAI) [18] and divided into 2 groups: stable patients (SLEDAI score < 5, *n* = 32) and active patients (SLEDAI score ≥ 5, *n* = 27), according to the physicians’ evaluation.

### Isolation of PBMCs and RNA extraction

Considering that autoreactive PBMCs, mainly lymphocytes, may participate in the autoimmune inflammatory process, we chose PBMCs as a source for determining MLKL mRNA level in SLE patients. The venous blood samples (4–5 mL) were collected in an EDTA-K2 tube from all the participants before breakfast, and PBMCs separated within 2 h by Ficoll (TBD Science, Tianjin, China) gradient centrifugation for 30 min at 1700 r/min. PBMCs were then transferred into 1 mL TRIzol Reagent in 1.5 mL centrifuge tubes and stored at − 80 °C until RNA extraction.

Total RNA was extracted from PBMCs by using TRIzol Reagent (Invitrogen, CA, USA) according to the manufacturer’s protocol and quantified with the NanoDrop ND-1000 (Thermo Scientific, USA). Approximately 200-800 ng of RNA was obtainted from 1mL of venous blood samples. Samples were used only if the ratio of the absorbance at 260 nm to that at 280 nm (A 260/A 280) was between 1.8 and 2.1. RNA samples with concentrations > 0.2 μg/μL were used for following reverse transcription reaction.

### Real-time polymerase chain reaction validations

According to the manufacturer’s recommendations, 20 μL of final reaction mixture was used containing 10 μL of SYBR Green PCR Master Mix (Takara, Dalian, China), 0.8 μL of sense primer, 0.8 μL of antisense primer, 0.4 μL ROX Reference Dye (50×), 6 μL of sterile deionized water, and 2.0 μL of the synthesized cDNA. Primers were designed by Primer Premier 5.0 and synthesized by Sangon Biotech (Sangon, Shanghai, China). Primers targeting MLKL and human 18S-rRNA were used—MLKL, forward: 5′-GCCACTGGAAAGATCCCGTT-3′, reverse: 5′-CAACAACTCGGGGCAATCCT-3′; human 18S-rRNA, forward: 5′-TGGAAATCCCATCACCATCTTCC-3′, reverse: 5-GGTTCACACCCATGACG-3′. The relative expression level of MLKL was normalized to the internal control 18S-rRNA expression and calculated by the comparative C_T_ (△△C_T_) method. Amplification was performed in 40 cycles (30 s at 95 °C, 5 s at 95 °C, 34 s at 60 °C) by ABI Step One Plus Real-Time PCR system (Applied Biosystems, CA, USA). A melt curve analysis was used to confirm the specificity of amplification.

### Serological assays

The serum total ANA was measured by an indirect immunofluorescence assay (Euroimmun, AG) with a titer of > 1:80 scored as positive. The antibodies to 15 antigens including double-stranded DNA (dsDNA), Smith antigen (Sm), and nucleosome (Nuc), SSA/60, SSA/52, SSB/La, ribonucleoprotein (rRNP), centromereprotein B (CENPB), ribosome P protein (Rib-p), histone (His), proliferating cell nuclear antigen (PCNA), Scl-70, Jo-1, and mitochondria (M2) were detected by chemiluminescent immunoassay (CLIA) (HOB, Suzhou, China). Serum complement 1q (C1q), complement 3 (C3), complement 4 (C4), immunoglobulin G (IgG), immunoglobulin M (IgM), and immunoglobulin A (IgA) were detected by immunoturbidimetric assay (Roche, Shanghai, China), and D-dimer concentration was determined with immunoturbidimetric assay (Sysmex, Japan) according to the manufacturer’s instructions.

### Statistical analysis

All data were statistically analyzed using GraphPad Prism 5 (version 5.0) software. Quantitative data were expressed as the mean ± SD. Data with a Gaussian distribution was analyzed using an unpaired *t* test or one-way analysis of variance (ANOVA), and Spearman’s rank was used to analyze the correlation of the numbers of leukocyte, lymphocyte, and monocyte, with the numbers of positive ANA, CRP, ESR, and D-dimer (D-D) levels. The area under the curve (AUC) was used to assess the specificity and sensitivity of using MLKL mRNA as a novel diagnostic tool for the detection of SLE. *p* values less than 0.05 were considered statistically significant.

## Results

### Characteristics and subgroups of SLE patients

Characteristics of the 59 SLE patients, 25 RA patients, and 30 matched HC individuals are shown in Table 1**.** The median age of SLE patients was 33.68 ± 13.55 years, with 96.6% females (57/59). The majority of SLE patients (48/59, 81.4%) were ANA-positive, less than half of the patients diagnosed with LN (23/59, 40.4%), 32 patients classified as stable patients (low disease activity), and 27 patients as active patients (high disease activity).

**Table 1:** Baseline characteristics of study groups

Variable	Groups		
	HC	RA	SLE
Patients/individuals (*n*)	30	25	59
Age (years), median (range)	32 (19–80)	30 (21–72)	34 (11–87)
Sex (F/M)	29/1	24/1	57/2
ANA test			
ANA+ (patients with positive ANA), *n* (%)			48 (81.4%)
ANA− (patients with negative ANA), *n* (%)			11 (18.6%)
Diagnosis based on renal involvement			
LN patients			23 (40.4%)
Non-LN patients			36 (59.6%)
Disease status			
Active patients (SLEDAI ≥ 5), *n* (%)			27 (45.8%)
Stable patients (SLEDAI < 5), *n* (%)			32 (54.2%)

### MLKL mRNA was upregulated in the PBMCs of SLE patients

**Fig. 1:**
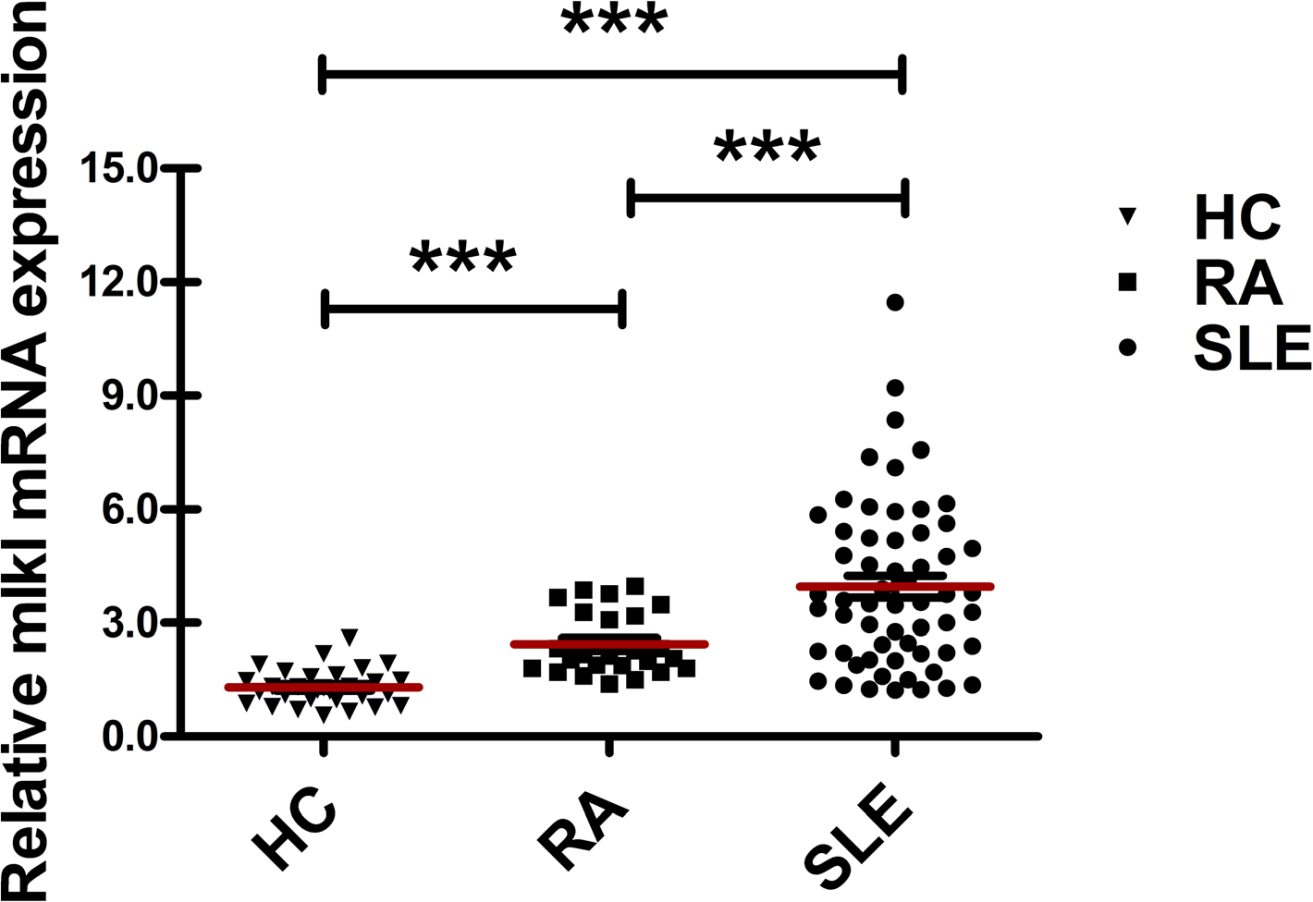
The increased levels of MLKL mRNA in the PBMCs of SLE patients. RT-PCR was used to determine the relative expression level of MLKL mRNA in the PBMCs of SLE (*n* = 59), RA (*n* = 25), and HC individuals (*n* = 30). ***p < 0.0001

We detected MLKL mRNA levels in the PBMCs of SLE patients, RA patients, and HC individuals. The levels of MLKL mRNA were significantly higher in SLE patients than in RA patients and HC individuals (*p* < 0.0001, respectively, Fig. 1).

**Fig. 2:**
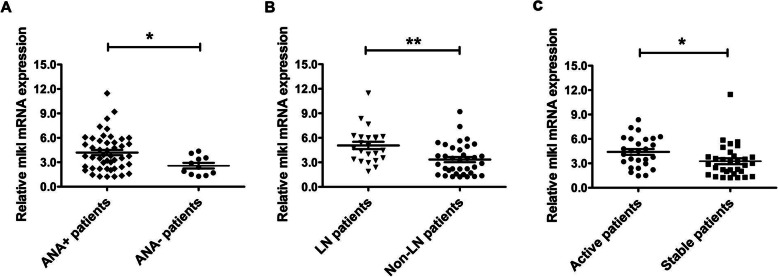
The differential expression of MLKL mRNAs in the three subgroups of SLE. **a** SLE patients with positive ANA (ANA+, *n* = 48) vs negative ANA (ANA−, *n* = 11). **b** LN patients (*n* = 23) vs non-LN patients (*n* = 36). **c** Active patients (SLEDAI score ≥ 5, *n* = 27) vs stable patients (SLEDAI score < 5, *n* = 32). *p < 0.05; **p < 0.005

We further analyzed the expression of MLKL mRNA in the subgroups of SLE patients and found that SLE patients positive for ANAs exhibited significantly higher levels of MLKL mRNA than those with negative ANAs (*p* < 0.05, Fig. 2a). MLKL mRNA was also found to be obviously upregulated in LN patients when compared with patients without LN (*p* < 0.005, Fig. 2b), and higher in active patients than in stable patients (*p* < 0.05, Fig. 2c).

### MLKL mRNA was positively correlated with clinical and pathological parameters of SLE

**Fig. 3:**
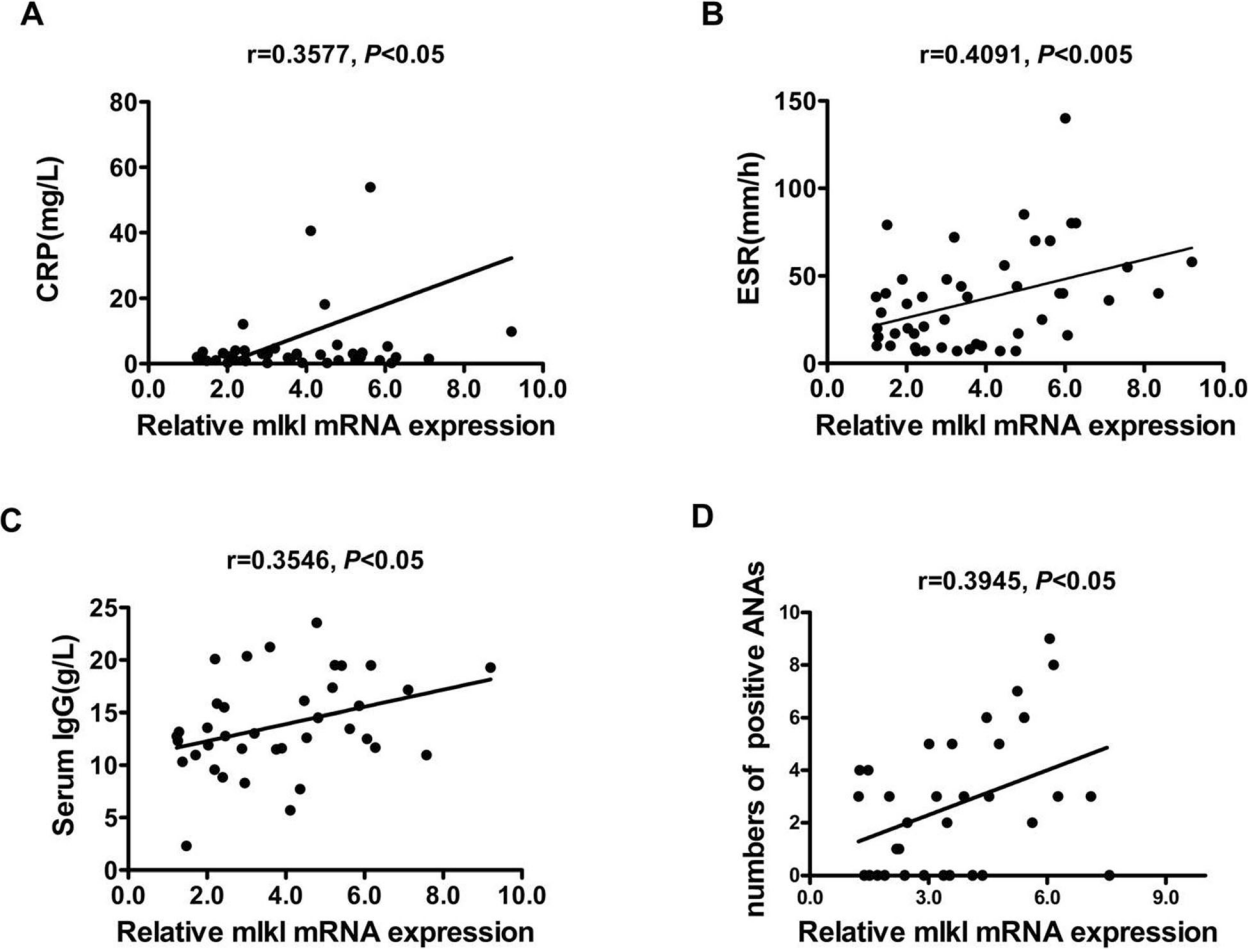
Positive correlations of MLKL mRNA levels with clinical parameters of SLE patients

The relationships between the levels of MLKL mRNA in PBMCs and the clinical or pathological characteristics of SLE are assessed and detailed in Table 2. Interestingly, MLKL mRNA level was significantly and positively correlated with ESR (*r* = 0.4091, *p* = 0.0043), CRP (*r* = 0.3571, *p* = 0.0237), serum IgG concentration (*r* = 0.3546, *p* = 0.0289), and the numbers of positive ANAs (*r* = 0.3597, *p* = 0.0432) (Fig. 3), but not associated with C3, C4, and other serologic indicators. Taken together, we found that increased MLKL mRNA was correlated with the activity in SLE patients.

**Table 2:** Association of MLKL mRNA with clinical pathological parameters of SLE

Clinical parameters	Data (mean ± SD)	Relative MLKL mRNA expression	
		***r***	***p***
Age (years)	33.68 ± 13.55	− 0.0132	0.346
ESR (mm/h)	36.10 ± 28.22	0.4091	**0.0043**
CRP (mg/L)	8.64 ± 24.03	0.3577	**0.0237**
IgG total (g/L)	13.80 ± 4.55	0.3546	**0.0289**
IgM total (g/L)	1.07 ± 0.49	− 0.1166	0.4919
IgA total (g/L)	2.59 ± 1.27	0.148	0.3821
C1q (ng/L)	177.80 ± 46.61	− 0.0421	0.8047
C3 (g/L)	4.92 ± 24.99	− 0.0439	0.7936
C4 (g/L)	0.18 ± 0.10	− 0.1836	0.2766
D-dimer (g/L)	397.4 ± 771.30	− 0.0569	0.7488
Leukocyte count (× 10^9^/L)	6.62 ± 2.68	− 0.0317	0.8382
Lymphocyte count (× 10^9^/L)	1.81 ± 0.72	0.1263	0.3819
Positive ANA numbers	2.78 ± 2.56	0.3945	**0.0432**
Anti-dsDNA antibody (IU/mL)	59.73 ± 113.3	0.1265	0.5214
Anti-Nuc antibody (RU/mL)	84.39 ± 139.60	0.0328	0.8656
Anti-Sm antibody (RU/mL)	41.75 ± 98.59	0.2085	0.3282

### MLKL mRNA in the PBMCs was sensitive for the diagnosis of SLE

**Fig. 4:**
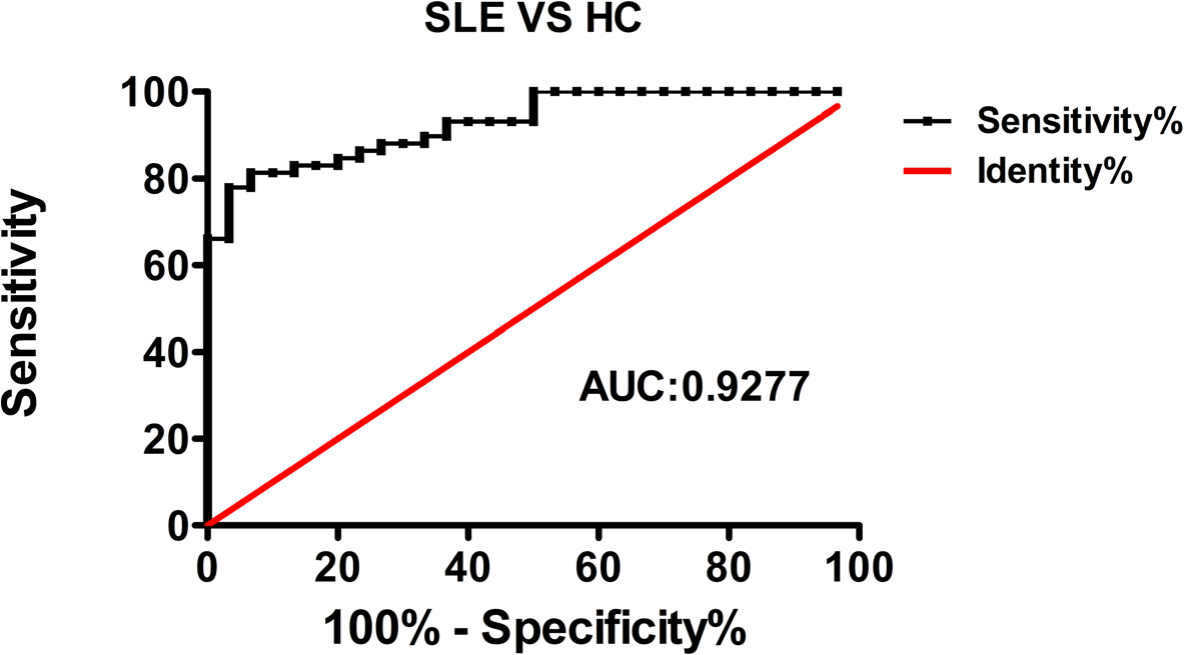
ROC analysis of MLKL mRNA for SLE patients

ROC analysis was employed to analyze the diagnostic efficiency of the MLKL mRNA for SLE patients. The diagnostic ability of MLKL mRNA achieved very high diagnostic accuracy 0.9277 (95% CI 0.878–0.978) with high sensitivity (81.36%) and specificity (93.3%), implying that MLKL mRNA of PBMCs may be a potential diagnosis biomarker for SLE (Fig. 4).

## Discussion

In this study, we showed that MLKL mRNA level in the PBMCs of SLE patients was significantly upregulated, especially in patients with positive serum ANAs. MLKL mRNA level in the PBMCs was also significantly and positively correlated with ESR (*r* = 0.4091, *p* = 0.0043), CRP (*r* = 0.3571, *p* = 0.0237), serum IgG concentration (*r* = 0.3546, *p* = 0.0289), and the numbers of positive ANAs (*r* = 0.3597, *p* = 0.0432). So far as we know, this is the first report that MLKL mRNA level in the PBMCs is increased in SLE patients.

Once phosphorylated, MLKL translocates from the cytosol to the plasma membrane to execute necroptosis. Defective clearance of necroptotic cells has been proposed to initiate inflammatory responses by the release of danger-associated molecular patterns (DAMPs). DNA acts as a major DAMP and is sensed in endolysosomes by toll-like receptor 9 (TLR9) and in the cytoplasm by cyclic GMP–AMP (cGAMP) synthase (cGAS), inducing the production of type I and type III IFNs and eliciting strong inflammatory responses [19, 20]. Several studies have demonstrated that patients with SLE have elevated circulating IFNs [21–23], whose signaling contributes to the steady-state expression of MLKL and the initiation of necroptosis, which not only causes tissue damage [6], but may also form a dynamic feedback loop in SLE pathogenesis.

Although SLE is a chronic inflammatory disease that can affect many organs, the kidneys are the mostly attacked [24]. LN is one of the most frequent and serious complications in SLE, and a real challenge for SLE treatment [25]. We surprisingly found that MLKL mRNA was obviously upregulated in the PBMCs of LN patients when compared with patients without LN (*p* < 0.005). To date, only one paper reported the correlation of necroptosis with LN, showing that PIPK3 and MLKL were activated in podocytes in renal biopsies from patients with LN [11]. Whether there is a crosstalk between the renal parenchymal cells and peripheral blood cells in necroptosis process still needs to be analyzed.

The current understanding of SLE implies autoimmunity to nuclear and cytoplasmic antigens, leading to generation large amount of ANAs [26]. Our findings are that SLE patients positive for ANAs exhibited higher MLKL mRNA levels than serum negative patients and that there are very significantly positive correlations between MLKL mRNA in the PBMCs and the numbers of positive ANAs or serum IgG concentrations, suggesting that necroptosis may play a potential role in the production of ANAs.

Conventional serologic ANAs are of limited sensitivity and/or specificity for diagnosis and monitoring in SLE [27]. Here, we reported that MLKL mRNA in the PBMCs could differentiate between SLE patients and HC individuals, and the AUC was as high as 0.9277 (95% CI 0.878–0.978) with high sensitivity (81.36%) and specificity (93.3%). As PBMCs are easy to obtain, this suggests that MLKL mRNA of PBMCs may be a novel biomarker for the diagnosis and monitoring of disease activity of SLE.

There were also several limitations. Firstly, as the patients in this study are from one hospital, whether there is a difference between patients from different areas is not known. Therefore, a multi-center cohort might be necessary for future implementation of techniques. Secondly, the molecular mechanism that how MLKL is involved in the progression of SLE remains unclear. Lastly, which specific cell of PBMCs expressed high MLKL mRNA level needs to be explored in the future.

## Conclusions

This is the first study to point out the upregulation of the MLKL mRNA in the PBMCs of SLE patients. The data presented here may provide certain evidence for the role of necroptosis in the pathogenesis and development of SLE, and also suggest new therapies by blocking signaling of necroptosis pathway in human SLE, especially in LN patients. Importantly, the MLKL mRNA expression levels in PBMCs may be useful in identifying those subgroups of SLE patients that may benefit from necroptotic blocking therapies. Finally, we believe that these findings could be of relevance for understanding the pathogenesis and diversity of SLE.

## Data Availability

The datasets generated and/or analyzed during the current study are available from the corresponding author on reasonable request.

## Abbreviations

MLKL: Mixed lineage kinase domain-like pseudokinase
PBMCs: Peripheral blood mononuclear cells
SLE: Systemic lupus erythematosus
RT-PCR: Real-time transcription-polymerase chain reaction
RA: Rheumatoid arthritis
HC: Healthy controls
ROC: Receiver operating characteristic
LN: Lupus nephritis
CRP: C-reaction protein
ESR: Erythrocyte sedimentation rate
ANAs: Antinuclear antibodies
IgG: Immunoglobulin G
IgM: Immunoglobulin M
IgA: Immunoglobulin A
CLIA: Chemiluminescent immunoassay
TNF: Tumor necrosis factor
TLR: Toll-like receptor
IFNs: Interferons
RIPK: Receptor-interacting serine/threonine-protein kinase
ACR: American College of Rheumatology
SLEDAI: SLE Disease Activity Index
DAMP: Damage-associated molecular pattern
cGAS: Cytoplasm by cyclic GMP–AMP (cGAMP) synthase
